# Practical Departments

**Published:** 1905-08-05

**Authors:** 


### PRACTICAL DEPARTMENTS.

### MESSRS. CLEMENT JEAKES AND CO.'S APPARATUS.

Messrs. Clement Jeakes and Co. have been makers of heating and similar apparatus for 120 years, and they make and fix all kinds of heating appliances, cooking apparatus, and laundry machinery. The firm make a rotary washing-machine 6 feet in diameter, capable of dealing with 300 shirts at once, and they claim to be the first makers of laundry machinery who applied reversing gear to the rotary-washer. The importance of the reversing gear is too well known to laundry engineers, and those who have anything to do with washing clothes to need description here. It will perhaps be sufficient to say that without the reversing gear the wear and tear, literally the latter, of clothes handled by rotary washing-machines would be very serious indeed, as the clothes work themselves up into a tangle that requires a great deal of undoing. The cage of Messrs. Jeakes's rotary washing-machine is constructed of iron or copper bars held by discs at the ends. The cases of the centrifugal wringing-machines made by the firm are of perforated sheet-copper, No. 10 gauge, the firm being of opinion that wire cages do not last, and that it is cheaper to fit a substantial cage at the beginning. In Calender machines they make apparatus up to 111 inches by 20 inches diameter, with machined bed, steam-heated. Messrs. Jeakes make ward-stoves for fixing at the ends of the wards, or in the centre, in which the air is heated on its way on to the ward by passing over the flue in which the hot gases from the fire are passing and with smoke flues leading upwards or downwards, the stoves being of all patterns commonly found in hospitals. In cooking appliances the firm make the usual steam-jacketed boiling pans and steam ovens, hot closets, carving tables, etc. The firm prefer to fit boiling pans and similar apparatus under a hood leading to a chimney, so that the steam shall pass up the chimney when the coppers or ovens are opened instead of out into the kitchen. Among other cooking apparatus Messrs. Jeakes fitted up the kitchens in connection with the Alexandra Trust; and at the Adelphi Hotel, Liverpool, the well-known stopping place for Atlantic travellers, they fitted up a combined cooking plant, comprising ovens, boilers, etc., measuring 20 feet by 6 feet, with a special arrangement for keeping soups hot at one end. Messrs. Jeakes fit up all kinds of heating appliances on the hot-water, live-steam, exhaust-steam, or vacuum systems, and they also fit up every kind of ventilating system. They have supplied and fitted hot-air apparatus for warming churches, and various other appliances.

